# Strengthening agroecological resilience through commons-based seed governance in the Philippines

**DOI:** 10.1007/s10668-022-02844-z

**Published:** 2022-12-21

**Authors:** Lea Kliem

**Affiliations:** 1 Department of Business Administration, Economics and Law, Carl von Ossietzky University, Oldenburg, Germany; 2 Institute for Ecological Economy Research, Berlin, Germany

**Keywords:** Agroecosystems, Commons, Resilience, Seeds, Governance, Philippines, Organic

## Abstract

The Filipino agricultural sector is exposed to multiple climatic, economic, and social risks that will likely intensify in the near future. Building agroecological resilience has been proposed to protect small-scale farmers’ livelihoods and improve food security in the context of (unexpected) shocks and disruptions, and slow system changes such as climate change. This paper argues that commons-based seed production, based on collective management and ownership of seeds and varieties, can play a central role in building resilience capacities in smallholder communities. I explore this by applying an indicator-based framework to assess the contribution of the Filipino farmer network Magsasaka at Siyentipiko para sa Pag-unlad ng Agrikultura (MASIPAG) to agroecological resilience. I find that the networks’ commons-based seed governance builds agroecological resilience in various ways. By equipping small-scale farmers with the tools to regain control over seed production and breeding, they become stewards of an actively evolving collection of varieties. The in situ maintenance and development of traditional, open-pollinated varieties and a network of diversified trial and backup farms build up buffering capacities and foster agrobiodiversity and local adaptation. A focus on regionally available natural resources reduces vulnerabilities to external factors. Adaptive capacities are strengthened through a high degree of flexibility and responsiveness achieved by self-organization and polycentric organizational structures. Broad participation, shared learning and spaces for experimentation support the development of farmers’ capacities to respond to diverse challenges. Commons-based approaches to seed governance can thus strengthen agroecological resilience and long-term food security in smallholder agricultural systems.

## Introduction

The Filipino agricultural sector is exposed to multiple interdependent climatic, economic, and social risks that will likely intensify in the near future (Eckstein et al., 2019; Yumal et al., 2011). Most prominently, the archipelago is highly vulnerable to climate change impacts. Increasing occurrences of super typhoons, floods, droughts, and landslides as well as changing rain patterns, and rising temperatures and sea levels directly impact Filipino agriculture (Hellin et al., 2020; Lansigan et al., 2000; Mallari, 2016; Stuecker et al., 2018). High levels of poverty among farmers also remain a pressing issue. With approximately one-third of the Filipino labour force working in the agricultural sector, over half of the population of 107 million depends, either directly or indirectly, on agricultural production (Davila, 2018; FAO, 2019). Ninety percent of Filipino farmers are small-scale farmers, who are particularly vulnerable since they often farm marginal land and have limited resources (Hellin et al., 2020; Lasco et al., 2016). Social and economic uncertainties fuelled by worsening inequalities, the COVID-19 pandemic, social unrest, and religious conflict also prevail, as agricultural reforms and market developments have failed to bring about trickle-down benefits (Reyes et al., 2012; Tuaño and Cruz, 2019). Moreover, the Philippines heavily depend on imports of rice, which is the country’s main staple food. This makes the Philippines vulnerable to global price fluctuations and market shocks (USDA, 2020). Achieving self-sufficiency in rice production thus remains a central goal of Filipino agricultural policy (Koirala et al., 2016).

The Philippines’ predominant approach to improving long-term food security in the face of external, unpredictable shocks and slow system changes, such as climate change and a continuously growing population, has been a strong focus on agricultural productivity and efficiency (Patel, 2013). The green revolution in the 1960s, with the International Rice Research Institute (IRRI) at the forefront of variety development, led to the rapid commercialization and intensification of agriculture based on generic high-yield varieties that are cultivated in monocultures and that are highly reliant on fossil fuel-based inputs (Harwood, 2019; Kastner, 2009; Kastner & Nonhebel, 2010; Stone & Glover, 2017). As a result of the increasing agricultural intensification and homogenization, agrobiodiversity strongly declined. Thousands of traditional rice varieties that had been cultivated for centuries, held cultural significance for local communities and were adapted to local environmental conditions, were replaced by a few modern high-yield varieties that are culturally and geographically disembedded (Montenegro de Wit, 2016; Patel, 2013; Stone & Glover, 2017). By 1970, 43% of rain-fed and 66% of irrigated agricultural land was cultivated with modern varieties. By 1985, the share had risen to 87% and 93%, respectively (Estudillo & Otsuka, 2006). Many of the over 5500 traditional Filipino rice varieties and the knowledge associated with their cultivation thus disappeared almost completely within a matter of years (Altoveros & Borromeo, 2007; Rosegrant & Sombilla, 2018).

Following increasing monopolization trends in the global seed sector, over half of the global seed market is controlled by only three technology-oriented agribusinesses that focus on high-yielding varieties primarily suited for large-scale industrial production. This trend leads to a lack of democratic participation, limited farmers’ access to diverse genetic resources, and a reduction of seed supply channels (Bonny, 2017; Howard, 2021). The narrow focus of these companies on genetically uniform varieties whose successful harvest depends on complementary agrochemicals, can lead to lock-in effects, and increase farmers’ dependence on agribusiness. Despite the failure of agricultural intensification to deliver positive social and environmental outcomes in low-income countries (e.g. Rasmussen et al., 2018), Filipino agricultural policies continue to be geared towards controlling environmental conditions through technological solutions that are often not suited for marginal land or come with high investment costs that are unaffordable for small-scale farmers. This is exemplified by the fact that the Philippines were the first county to approve genetically modified “Golden Rice” in July 2021. Golden rice is genetically engineered to produce beta-carotene in the edible part of the plant, to address Vitamin A deficiencies in the Filipino population. Focusing on expensive technical solutions, rather than supporting the consumption of naturally vitamin A rich foods, comes at the expense of locally adapted, traditional rice varieties that are closely connected to Filipino cultural heritage and cuisine. Moreover, traditional varieties are often tightly interwoven with traditional agricultural practices and intangible ecosystem services such as cultural and ecological knowledge and sociocultural values (Pfeiffer et al. 2006; Tekken et al., 2017).

To conserve and enhance agrobiodiversity, protect small-scale farmers’ livelihoods, and improve food security in the context of (unexpected) climatic, economic, and social shocks and disruptions, building resilience has been proposed by scholars and (inter)national policy actors alike (Darnhofer et al., 2010; FAO et al., 2018; Hellin et al., 2020; Schipanski et al., 2016; Seekell et al., 2017). Resilience refers to the emergent properties that contribute to social-ecological systems’ ability to withstand (unforeseen) disturbances, both through buffering shocks and adaptation (Carpenter et al. 2001). Given that the trajectories of social-ecological systems can never be fully predicted, using resilience as an analytical framework can help to shift the focus from short-term optimal solutions to long-term sustainable transition paths (Darnhofer, 2014; Hodbod & Eakin, 2015). By acknowledging the complexity and uncertainty of the future, resilience approaches highlight the potential for adaptation in continuously changing social-ecological systems. In the context of agricultural production, resilience refers to the capacity of agricultural systems to ensure long-term food security in case of perturbations. Much literature has focused on identifying resilience properties in the agricultural sector. Although seed production and breeding play a central role in building resilience, the governance of genetic resources, including breeding and seed production, has received limited attention in this context (Lammerts van Bueren et al., 2018). Previous research suggests that a commons orientation in seed production, based on collective management and ownership, including the participation of smallholder farmers in variety development, is particularly suited to strengthen agroecological resilience (Kliem & Sievers-Glotzbach, 2021). This paper investigates this further, through an in-depth case study of the farmer-led Filipino network Magsasaka at Siyentipiko para sa Pag-unlad ng Agrikultura (MASIPAG) which translates to Farmer-Scientist Partnership for Development.

The study aims to explore to what extent and how MASIPAG’s commons-based approach to breeding, seed production, and seed handling contributes to agroecological resilience in the Philippines. Previous research has examined MASIPAG’s technical efficiency (Velasco 2019), as well as the network’s contribution to intra- and intergenerational environmental justice (Sievers-Glotzbach, 2014), food security and food sovereignty (Bachmann et al., 2009; Heckelman & Wittman, 2015), farmers’ empowerment (Bachmann et al., 2009) and knowledge production (Frossard, 2002). Most relevantly, Heckelman et al. (2018) examine MASIPAG’s contribution to climate change resilience in comparison to conventional agriculture in the province of Negros Occidental using the United Nations Food and Agriculture Organisation’s Self-evaluation and Holistic Assessment of Climate Resilience of Farmers and Pastoralists (SHARP) tool. Building upon this research, this paper specifically focuses on breeding and seed production. It sheds light on the organizational structure, principles and practices that enable MASIPAG to achieve its breeding and conservation goals. Furthermore, it assesses the network’s agroecological resilience capacities by applying an indicator-based analysis framework. A particular focus is placed on examining the influence of the network’s commons-based governance arrangements on agroecological resilience. The paper thereby leads to a better understanding of the relationship between commons-based breeding, seed production and agroecological resilience. By drawing on a specific case study, the research complements more conceptual studies on agroecological resilience that focus on the exploration of broader social-ecological system dynamics (e.g. Rotz and Fraser, 2015; Urruty et al., 2016; Walsh-Dilley et al., 2016).

First, I outline the relevance of seed production and breeding for agroecological resilience and specify the conceptual framework used for the analysis. Next, I describe the research design and methods. The subsequent section presents and discusses the results. Finally, I summarize the findings and place them in the context of broader discussions on the relationship between commons-based seed governance, smallholder resilience and food and seed sovereignty.

## Conceptual framework

### Agroecological resilience and seed production and governance

The concept of resilience [from the Latin word verb resilire or “leaping/bouncing back”] originates in the field of ecology where it refers to the stability of ecological systems in the context of disturbances (Holling, 1973, 1996). In recent years, it has been broadened in its scope of application and is now used across natural and social disciplines to study social-ecological system dynamics (Beichller et al., 2014; Moser et al., 2019). Such social-ecological or human ecology-based analyses provide insights into the interactions between social and environmental system components and allow for the identification of dominant paradigms that shape food and agricultural systems (Davila, 2018; Dyball et al., 2020). As a conceptual lens, resilience is hence useful for examining pathways that challenge predominant agricultural development paradigms, which are often inclined to business-centric models, based on output maximation and technocratic approaches that focus on modernization through new technologies, crops, and farm inputs (Chaudhuri et al., 2021).

Several studies have used resilience as a conceptual lens to (i) study the social-ecological interrelations and dynamics of agroecosystems in times of disturbance and/or (ii) examine factors contributing to resilience in agricultural systems. Altieri et al. (2015) and El Chami et al. (2020) for example emphasize the relevance of agroecology and high levels of biodiversity for increasing the resilience of agricultural systems in the face of climate change. Similarly, Urruty et al. (2016) examine the concepts of stability, robustness, vulnerability, and resilience and conclude that increasing diversity and adaptive capacity bear the largest potential for enabling agricultural systems to withstand extreme and unpredictable changes. Darnhofer et al. (2010) and Darnhofer (2014) outline how resilience thinking can help to better understand the interdependence of social and ecological systems to increase sustainability on the farm level. Rotz and Fraser (2015) take a historical perspective and explain how the social and technical developments of the industrialization have diminished resilience in the agricultural sector, while Sinclair et al. (2014) explore the usefulness of resilience thinking for contemporary agricultural transformations. Tendall et al. (2015) and Hodbod and Eakin (2015) broaden the scope of analysis and use a resilience approach to highlight the complexity and multifunctionality of entire food systems. Building on this, Schipanski et al. (2016) identify strategies to foster food system resilience across different scales.

The focus of these and other studies is often primarily on agricultural production and crop cultivation, with little attention given to earlier stages of the value chain (Lammerts van Bueren et al., 2018; McGuire & Sperling, 2013; Pautasso et al., 2013). Yet, there are several reasons to assume that seed production, including breeding, is important for building resilience in agricultural systems: (i) Seeds are the foundation of all agricultural production. Disturbances related to seed production hence possibly impact the entire agricultural value chain. (ii) Varieties’ agroeconomic characteristics impact their suitability for certain environmental conditions. Breeding thus affects the adaptability of agricultural systems to changing climatic and biophysical conditions. The dominant agricultural paradigm, driven by economic interests and the commodification of agricultural inputs, suggests that environmental influences lose relevance since they can be increasingly controlled and optimized. However, under less than optimal growing conditions, which will likely increase in the face of climate change and biodiversity loss, adaptation to local environmental conditions is of crucial importance to ensure stable production and ecosystem service provision (Ficiciyan et al., 2018). Furthermore, the stability of agricultural systems is directly impacted by the diversity within and between varieties. Genetic diversity is thus a prerequisite for long-term food security (Jackson et al., 2007; Lammerts van Bueren et al., 2018; Thrupp, 2000). (iii) The governance of genetic resources is linked to questions concerning the access and ownership of seeds and varieties, which can impact farmers’ resilience capacities. In specific, global intellectual property rights, international treaties, and national regulations on variety registration, ownership and use, affect the availability and access of varieties. Following large-scale mergers of seed companies in recent years, the intellectual property rights for the genetic basis of our global food supply are increasingly in the hands of only a few multinational cooperations that are driven by economic interests. This has resulted in a narrowing of the genetic range to a few varieties that are particularly profitable to these cooperations but leave limited choices for farmers that are dependent on their assortment (Aguilar, 2001; Clancy & Moschini, 2017). Intellectual property rights also limit farmers’ and breeders’ access to breeding material, which curtails the development of new, locally adapted varieties. It has thus been proposed that alternative organizational arrangements where the de facto handling, breeding, and sharing of seeds and varieties are carried out collectively by a community of farmers and breeders may contribute to building agroecological resilience capacities (Kliem & Sievers-Glotzbach, 2021). Such commons-based governance arrangements are characterized by (i) the collective responsibility for the provision and development of seeds and crop diversity, (ii) the protection of seeds from legal and bio-technological enclosure, (iii) polycentric management structures, defined as multiple, formally independent centres of decision-making that collectively manage seeds and (iv) the sharing of formal and practical knowledge within the seed initiatives and beyond (Sievers-Glotzbach et al., 2020).

### Agroecological resilience assessment

The multidimensional nature of resilience and the complex dynamics of socio-ecological systems make the assessment of resilience challenging (Quinlan et al., 2016). Several frameworks have emerged to operationalize the assessment of agroecological resilience (Altieri et al., 2015; Cabell & Oelofse, 2012; Green et al., 2017; Seekell et al., 2017; Tittonell, 2020; Worstell & Green, 2017). The most frequently cited framework was compiled by Cabell and Oelofse (2012), based on a literature review. It encompasses 13 indicators, related to various social-ecological dimensions, whose presence indicates the capacity of agricultural systems to buffer, adapt or transform in case of disturbance. The framework has been developed further by Kliem and Sievers-Glotzbach (2021) to address resilience dimensions that are particularly relevant in the context of seed production and breeding. For this paper, the framework by Kliem and Sievers-Glotzbach is used to assess MASIPAG’s contribution to agroecological resilience (see Table 1). The framework covers aspects of both, the system-to-be-governed (the seed system and its linkages to plant cultivation) and the governance system (the institutions, values, rules and economic incentives impacting breeding and seed production).

**Table 1:** Agroecological resilience assessment framework by Kliem and Sievers-Glotzbach (2021).

Indicator for agroecological resilience	Description of the indicator concerning seed production and breeding
Socially self-organized	The social components of the agroecosystem can form their own breeding and seed production structures that fit specific needs (e.g. of organic or small-scale agriculture)
Ecologically self-regulated	Availability of naturally reproducible varieties that can adapt to changing local conditions, including ecological pest management
Functional and response diversity	Breeding and seed multiplication approaches that foster agrobiodiversity at the level of genetic diversity, crop species, and landscapes to increase functional and response diversity in plant cultivation
Optimally redundant and accumulation of reserves and physical infrastructure^a^	Redundancy of different supply channels for seeds and diversity of breeding organizations and structures. Physical facilities for breeding, seed processing and storage are build up and maintained on-farm. Seed banks preserve genetic resources in the long term
Exposed to disturbance	Variety development takes place under careful exposure to disturbance through in situ breeding processes
Coupled with local natural capital	Availability of varieties that are adapted to regional environmental conditions and resources. Focus on varieties that are based on the bioregionally available natural resources
Appropriately connected: globally autonomous and locally interdependent in modular subsystems	Reduced reliance of (new) varieties on external inputs such as pesticides, fertilizers, and international patents. A high degree of modular connectedness at the local level of breeders, farmers, gardeners, and other actors along the value chain
Builds human capital and encourages reflective and shared learning	Skills, knowledge and expertise on breeding and seed production are build up and social networks are formed. Knowledge and skills on breeding and seed multiplication as well as on variety traits and cultivation are documented, disclosed, and passed on. Practical on-farm experience is integrated into breeding activities
Conservative innovation that honours legacy	Use of traditional breeding methods (besides some modern breeding techniques) for the continuous development of new varieties. Ensuring the availability and use of underutilized and heirloom varieties
Reasonably profitable	Long-term financial viability of breeding and seed multiplication is ensured
Bases on polycentric, decentralized governance structures	Breeding and seed production is organized through decentralized governance structures at various levels
Ensures resource access and broadens participation	Access to seeds and genetic material, including for small-scale farmers, gardeners, and breeders, is ensured. Broad participation in breeding and conservation efforts is given. Decision-making processes within organizations are inclusive
Encourages complex adaptive systems thinking	Holistic approaches to breeding and seed production acknowledge the interdependencies of social and ecological factors and their partial unpredictability

^a^In this paper, the indicators *optimally redundant* and a*ccumulation of reserves and physical infrastructure* were merged, since they address overlapping aspects relating to the redundancy of available resources

## Research design

A case study approach was chosen to provide an in-depth exploration of MASIPAG’s practices. Compared to other research designs, case studies allow for the collection and analysis of data that is richer and of greater depth. In the context of the Philippines, MASIPAG provides a particularly interesting example for exploring long-term seed governance practices.

### The farmers’ network MASIPAG

Originally a small-scale breeding programme aiming to conserve and improve native rice varieties, the farmer-led Filipino network MASIPAG organized in 1986 in response to the green revolution. It has since developed into a nationwide movement that has worked with over 35,000 farmers, 60 non-governmental organizations and 15 scientific partners. The network’s primary aim is to secure resource-poor farmers’ livelihoods by promoting “sustainable use and management of biodiversity through farmers’ control of genetic and biological resources, agricultural production and associated knowledge” (MASIPAG, 2013, para. (1). It does so by focusing on the collective management, maintenance, and exchange of seeds to ensure the in situ conservation of locally adapted rice varieties (Sievers-Glotzbach et al., 2020, 2021).

### Data collection and analysis

This analysis is based on semi-structured interviews with 12 key informants. The interviews took place in Nueva Ecija in Central Luzon—the “rice bowl” of the Philippines—in February 2019. Key informants included members of the MASIPAG Board of Trustees, national, regional and provincial staff, staff from the national backup farm and MASIPAG farmer-leaders from Luzon, die Visayas and Mindanao. Interviewees were identified through purposive snowball sampling, in order to interview members of the network that cover a wide range of positions and perspectives. Interview questions related to (i) the principles of the network concerning seed handling, seed production and breeding, (ii) the practical implementation and practices regarding breeding programmes, seed multiplication and dissemination, and (iii) the governance and organizational structure of the network. The questions covered aspects relating to all 13 indicators. Interviews lasted from 80 to 160 min. They were carried out in English since the majority of interviewees were fluent in English. When this was not the case, a local Tagalog-English translator supported the interview process.

All interviews were fully transcribed and coded using the program MAXQDA. In total, 721 interview segments of varying lengths were identified as relevant. Each text segment was assigned to one or several resilience indicators. The subsequent qualitative content analysis, following Kuckartz (2016), focused on identifying reoccurring themes and statements that provide information serving the evaluation of each indicator. The focus of the analysis was on the development, production, and handling of seeds. Yet, in some respects, these are inseparable from general farming practices or larger organizational structures. Where this is the case, these were taken into consideration.

The interviews were supplemented with data from participant observations at a weeklong workshop with MASIPAG staff and farmers, which took place at MASIPAG’s national backup farm in Nueva Ecija in Central Luzon in February 2019. The detailed notes that were taken during this event inform the analysis and substantiate the discussion were applicable.

## Results and discussion

The analysis shows that MASIPAG’s approach to seed production and breeding contributes to agroecological resilience in the Philippines in various ways. In the following, I discuss each of the 13 indicators and, where applicable, place them in the context of relevant studies. At the end of each indicator, I draw a short resume if and how MASIPAG’s approach contributes to agroecological resilience.

### Socially self-organized

**Fig. 1:**
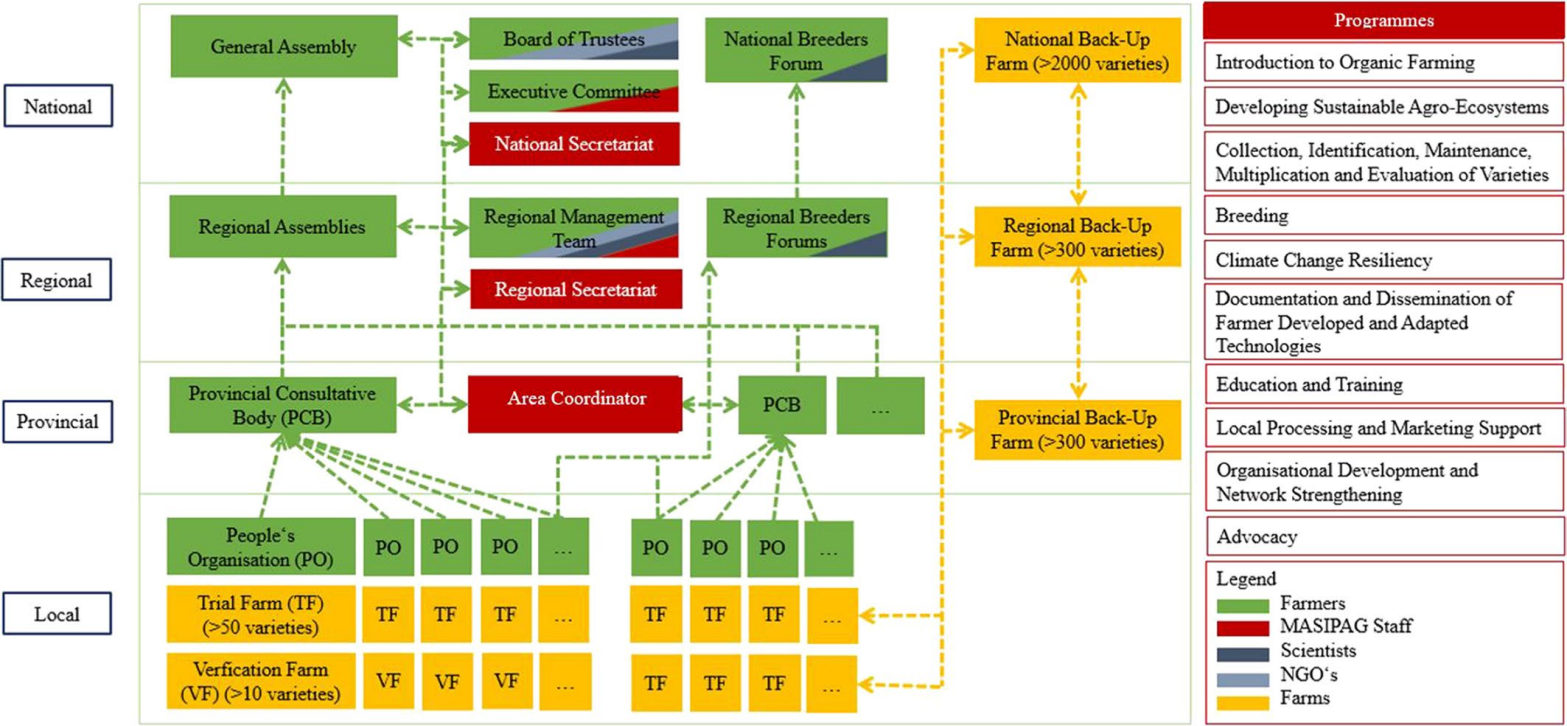
MASIPAG’s organizational structure

Social self-organization is a key property of resilient systems, with bottom-up initiatives and local and regional networks often being more responsive and adaptable to changing conditions than top-down initiatives (Cabell & Olefse, 2012; Mukhovi et al., 2020). Self-organization is ingrained in MASIPAG’s organizational structure, which comprises 635 self-governed, independent farmer associations. Groups of typically 10–15, sometimes up to 50, rice and vegetable farmers form the basic organizational unit of MASIPAG (see Fig. 1). These farmer groups are referred to as People’s Organisations (POs). POs are financially independent and provide platforms that enable their members to set common goals and coordinate their farming, seed production and breeding efforts. They act as social insurance that allows farmers to rely on mutual support in times of hardship and (unexpected) disturbance. Through work-share practices and joint planning, collective responses to disturbances and preparations for potential calamities and threats are coordinated. Individual farmers cannot join MASIPAG unless they have organized themselves into a local PO. Self-organization is thus a prerequisite for the initiative’s bottom-up approach. A staff member described:

We rely on independent farmer groups. We call them POs. […] The POs’ have different organisational structures and we respect these different structures. However, we educate them about organisational policies. They should not be leader-centred. Decision-making should not come from the leaders only but should be exercised by the whole [group].[…] There is mutual support, […] somehow it’s like a social insurance. For other members to be there to help whenever they are in distress […] So there is a sense of community: Helping one another and belonging and that’s important [Interviewee #3].

MASIPAG’s focus on self-organization weaves through all organizational levels of the network. On the provincial level, Provincial Consultative Bodies (PCBs), comprised of representatives of all POs in the region, decide on strategic matters, coordinate trainings and activities, monitor progress and act as conflict mediators. Self-formed technical committees (e.g. on climate change resilience, breeding or organizational development) at the local and provincial levels allow for distributing responsibilities and sharing skills and knowledge. MASIPAG’s scientific and NGO members support committees on an ad-hoc basis as needed. On the regional and national levels, farmer assemblies coordinate programming and decide on long-term strategic matters. The network’s 40 staff members that work as local area coordinators or at national and regional secretariats primarily act as facilitators that support the development and implementation of farmer-led programmes rather than as programme initiators.

This farmer-centred, bottom-up approach fosters a high degree of self-organization among the network’s members and may contribute to agroecological resilience by enabling a high degree of flexibility and responsiveness to local needs. Working together can help farmers to tolerate, cope with, and adjust to environmental and social stresses and lessen the burden they would face individually (Mukhovi et al., 2020). It also contributes to building a collective identity (López et al., 2019). However, self-organization presupposes a high level of engagement of farmers in communal activities, and some interviewees reported that a lack of commitment from individual farmers weakens social cohesion. A high level of self-organization also makes the network vulnerable to conflicts and internal governance issues. Farmers’ interests and motivations are heterogenous and can lead to disputes that fragment norms and practices (Sherwood et al., 2016). Non-cooperation between farmers which results from conflict or disputes, may also weaken social ties and ultimately hamper resilience capacities.

### Ecologically self-regulated

Self-regulating agroecosystems provide ecological feedback mechanisms that increase systems’ responsiveness and thus their capability to adapt to both internal and external changes (Cabell & Oelofse, 2012; Worstell & Green, 2017). MASIPAG fosters ecological self-regulation in various ways. The initiative places a strong focus on organic farming and breeding, which has been found to contribute to building agroecological resilience (Borron, 2006; Carpenter, 2003; Kummer et al., 2012; Lammerts van Buerren et al., 2002). All breeding, seed multiplication and cultivation of MASIPAG farmers takes place under organic conditions. One farmer explained: “We do it all organically, so we do not cause harm to the environment. We only use the natural stuff” [Interviewee #8]. To become a member of the network, farmers must commit to eliminating chemical inputs and work towards converting to organic agriculture standards that are equivalent to those of the International Federation of Organic Agriculture Movements (IFOAM). Compared to conventional Filipino farmers, MASIPAG farmers use fewer chemical pesticides and apply natural fertilizers made from local ingredients, thereby contributing to the maintenance of local regulating ecosystem services. This observation has also been made by Bachmann et al. (2009) and Heckelman et al. (2018). Farmers’ compliance with organic standards is continuously inspected and monitored by community members through the network’s own participatory guarantee system, which 47 of MASIPAG’s POs have joined.

Furthermore, (new) varieties are specifically bred to meet the needs of organic agriculture and are selected to adapt to local environmental pressures such as low-fertility soils or potential climate change threats. Agronomic characteristics supporting resilience properties such as salt-water adaptation, drought tolerance, or resistance to pests and diseases are given equal consideration in variety development as breeding goals relating to yield. This ensures optimal adaptation to constantly changing environmental conditions and marginal lands. MASIPAG has developed 12 flood-tolerant, 18 drought-tolerant, 20 salt-water tolerant and 24 pest-resilient varieties that are adapted to various Filipino regions. The network has also identified numerous traditional varieties that possess these qualities.

For both cultivation and breeding, MASIPAG members rely on open-pollinated, naturally reproducible varieties. Varieties that are not self-seeding such as hybrids or genetically modified varieties, which are actively promoted by the Filipino government (c.f. Glover et al., 2020), are rejected. The reliance on open-pollinated varieties allows farmers to harvest seeds and save them for the next planting season, which contributes to the continuous local adaptation of varieties. In comparison to hybrid varieties, open-pollinated varieties often have lower yields when planted in optimal or near-optimal environmental conditions (e.g. Peng & Kush, 2003). Yet, studies show that they can outperform hybrid varieties in harsh environmental conditions (e.g. Ficiciyan et al., 2018), which is the reality for many Filipino small-scale farmers who frequently work on marginal land. Furthermore, resource-poor farmers can often not afford the fertilizer application rates needed to achieve the maximum yields of hybrid varieties. MASIPAG’s focus on organic agriculture practices and their reliance on open-pollinated, locally adapted, robust varieties, hence contributes to agroecological resilience by building farming systems that can regulate naturally to changing environmental and climatic conditions.

### Functional and response diversity

Building agricultural systems around agrobiodiversity has been shown to foster resilience (Dwivedi et al., 2017; Jackson et al., 2007; Lin, 2011; Mijatović et al., 2013). Diversity prevents total system collapse since diversified farming systems have lower susceptibilities to any particular environmental pressure (Cabell and Oelofse, 2012; Darnhofer et al., 2010; Stockholm Resilience Centre, 2015). MASIPAG prioritizes the conservation and cultivation of agrobiodiversity at the genetic, species and landscape level. At the genetic level, breeding open-pollinated varieties through bulk selection results in varieties with a broad genetic basis that stand in contrast to genetically narrow inbred lines of commercial breeders. Bulk selection is the practice of planting entire populations and selecting single plants only in later generations. It is a simple and practical breeding method for generating phenotypically uniform but genetically diverse varieties that allows for natural selection to occur during the advance of generations and can easily be carried out on-farm (Corte et al., 2002). It is thus suitable for cost-effectively handling maximum genetic diversity (Das, 2018).

MASIPAG also fosters diversity by continuously cultivating over 2000 rice varieties at both of their national backup farms, and over 300 rice varieties at each of their eight regional and provincial backup farms (see Fig. 1). The purpose of these backup farms, which are run by MASIPAG staff, is to ensure that all of the networks’ varieties are constantly cultivated and available. To date, MASIPAG has collected 1313 traditional rice varieties and bred 1299 new varieties, 506 of which were bred by farmers. The network has also collected 105 traditional corn varieties, which continue to be developed, as well as a range of vegetable varieties including eggplant, beans, squash, peppers and tomatoes. One farmer described:

MASIPAG […] give us the culture that wherever you go, [you] gather seeds. Of course, with the consent of the other farmers […] Farmers that we are networked with, they learned that we have this culture of gathering seeds of different kinds, not only rice but also vegetables. If they have a new variety, they want to give it to us for preservation. [Otherwise, when] disaster comes and they lose the seeds, they will not be available for them - they will lose the genetic resources. So, we've learned that the more you share, the more you [can] secure [the] genetics of the seeds [Interviewee #10].

MASIPAG’s backup farms are supplemented by a network of around 190 trial farms, spread around the country. In their founding phase, each PO sets up a trial farm to determine which varieties are best suited for their local particularities. Each newly established trial farm is supplied with small seed quantities of a random selection of 50 rice varieties from one of the backup farms. Out of these, POs choose a selection of around ten of the best-suited varieties to be trialled on so-called verification farms. They subsequently select three to five varieties for mass production. To support POs in their process of further diversification, they are periodically supplied with an additional set of 50 randomly chosen varieties. This elaborate setup ensures that a diverse range of varieties is continuously cultivated across all regions and that new genetic material is consistently made available for breeding and local adaptation purposes.

At the landscape level, MASPAG farmers engage in a range of diversification practices. According to Bachmann et al. (2009), they have a higher crop and livestock diversity than conventional farmers, with an average of 45 different crops, compared to 30 different crops grown by conventional farmers. This diversification reduces the risk of a full crop failure. In addition to their main crops, MASIPAG farmers often plant “survival crops” such as root crops that are not as badly affected by typhoons. Survival crops allow farmers to continue their farm operations and seed work in case of calamities. Many MASIPAG farmers also engage in staggered planting dates, which ensure continuous seed and crop production throughout the year and decrease the likelihood of total harvest loss through minimizing pest infestations (c.f. Carpenter, 2003). MASIPAG farmers also employ more diverse pest and animal disease control methods than conventional farmers (Heckelman et al., 2018). Hence, MASIPAG’s focus on genetic diversity, its nationwide network of trial and backup farms and farmers’ diversification practices, foster agroecological resilience by securing agrobiodiversity and spreading the risk posed by disturbances.

### Optimally redundant and accumulation of reserves and physical infrastructure

Redundancy allows for back-ups that can support the buffering of shocks but may reduce efficiency due to resources invested in building-up capacities that may never be used (Cabell and Oelofse, 2012; Stockholm Resilience Centre, 2015). MASIPAG creates redundancy of seeds and genetic resources in various ways. Its network of trial and backup farms serves as an in situ seed bank. It ensures that varieties are continuously cultivated and saved at multiple locations across the country. In addition, farmers are encouraged to create redundancy at the farm level, by building up emergency seed reserves for times of disturbance and harvest failure. One farmer stated: “We always […] ensure that we have reserves. […] The typhoon affects me, but we prepare. That is my resilience, how I prepare. Disaster is anytime anywhere, so I need to prepare for that, anticipate. If flooding happens or if my crops are destroyed, I should have stored extra seeds to maintain” [Interviewee #10]. Redundancy is thus encouraged at both, the farm and the organizational level. However, it was reported that not all POs have seed reserves and that backup mechanisms may not function as well if entire regions are affected by calamities and local support through other POs is limited.

MASIPAG also contributes to the redundancy and diversification of seed supply channels through seed-sharing practices within and beyond the network (see Sect. 4.7, 4.12). Yet, according to Heckelman et al. (2018), the diversity of sources of seed acquisition of individual farmers is not higher than that of conventional farmers, supporting the finding that MASIPAG farmers primarily obtain their seeds from within the network. Overall, MASIPAG’s redundancy in variety trailing and the network’s (emergency) seed reserves contribute to agroecological resilience by creating fallback options that have proven useful to farmers during times of calamities and other disturbances.

### Exposed to disturbance

Careful exposure to disturbance increases agroecological resilience by allowing systems to develop coping mechanisms and recover from disruption (Cabell & Oelofse, 2012; Worstell & Green, 2017). MASIPAG exposes varieties to disturbance through on-farm, in situ breeding, as opposed to breeding under artificial laboratory conditions. The networks’ field-based farmer laboratories, allow farmer-breeders to observe varieties under messy and complex real-life conditions and select traits that are particularly relevant to them. This approach enables MASIPAG to select varieties even in the remotest locations. Such in situ breeding increases the likelihood of new genetic combinations and allows plants to adapt to their biotic and abiotic environments (Bellon et al., 2017). The resulting site-specific varieties are thus optimally adapted to the biophysical conditions they were exposed to during selection. A staff member described:

All of the rice collection of MASIPAG is what [we] call a locally specific variety. […] For example, M45 is a product of rice breeding of MASIPAG. When we planted it here in Nueva Ecija, we identified that this variety is a draught resilient variety. But when we brought it to other regions like southern Luzon, some farmers identified this variety [as] tolerant to seawater intrusion. What I want to emphasize is all of our varieties [are] locally adapted. That is one of the ideas of the trial farm. We distribute 50 varieties, so that among them the farmers can choose which varieties [are] most adapted to their place [Interviewee #1].

In contrast to ex situ conservation in frozen seed vaults, which bear the dangers of defrosting, declining seed viability, and loss of associated knowledge, the in situ maintenance of varieties through cultivation provides long-term biological insurance and allows for the continuous co-evolution of knowledge and genetic material. Although both types of conservation play a central role in biodiversity protection, in situ conservation provides the best long-term option for conserving genetic diversity (Mestanza-Ramón et al., 2020). Hence, MASIPAG’s in situ breeding and conservation contribute to agroecological resilience by allowing varieties to develop robustness through exposure to local environmental pressures.

Concerning exposures to disturbances such as typhoons or unusual pest infestation, Heckelman et al. (2018) find no differences between MASIPAG and conventional farmers. Nevertheless, despite disaster preparations, many of the interviewed farmers identified climate risks including exposure to typhoons and pests as the single largest threat to their livelihoods.

### Coupled with local natural capital

Agroecosystems aligned with local natural capital encourage the responsible use of local resources to ensure their long-term availability (Cabell and Oelofse, 2012; Darnhofer et al., 2010). MASIPAG is highly reliant on local natural resources. Farmers are strongly encouraged to produce their own farm inputs, including planting materials, organic fertilizers and botanical sprays required for seed production and plant cultivation. Where possible, farmers make use of freely available local materials. One farmer described: “We use what we have available here. Fermented plant juice against pests, green manure, rice straw as fertilizers and so on. There is so much you can use if you know how to do it” [Interviewee #7]. Their reliance on local resources is primarily motivated by the network’s strive towards complete independence from (international) agribusiness (see Sect. 4.7). This reduces farmers’ financial vulnerability since lower economic investments are required in the first place. Furthermore, as previously described, MASIPAG relies on local, traditional varieties and trials new varieties based on their adaptation to local environmental conditions, thereby directly connecting them to bioregionally available resources. MASIPAG’s reliance on locally derived farm inputs thus positively affects agroecological resilience, by aligning seed production to operate within the means of the local ecosystem. Heavy reliance on local resources, however, also increases vulnerabilities in case of resource depletion that is beyond the networks’ control. For instance, if local forests or water resources are not managed sustainably, agricultural inputs may no longer be available and ultimately threaten food production (c.f. Briones, 2005). None of the interviewees addressed such issues, but since regional resource management is beyond the network’s scope of influence, it may pose a threat to the network’s long-term work.

### Appropriately connected: globally autonomous and locally interdependent

Global autonomy increases freedom from external uncontrollable developments, whereas local interdependence fosters trust and collaboration (Cabell & Oelofse, 2012; Stockholm Resilience Centre, 2015). MASIPAG farmers have a relatively high level of local connectedness and strive for global autonomy. A board member stated: “We struggle against the powers of corporate control in food and agriculture. [So] we choose to have our own seeds, planting materials, fertilizers, [and] natural pesticides. We are independent and autonomous” [Interviewee #2]. The network fosters a strong culture of local seed sharing, with over three-quarters of MASIPAG farmers engaging in seed exchange practices. This observation has also been made by Bachmann et al. (2009). The free exchange of seeds is a crucial element and ritual practice at almost all of MASIPAG’s meetings, leading to a continuous flow of seeds within the MASIPAG community. Local connectivity is also fostered through farmers’ pooling of resources and their reliance on the traditional practice of Bayanihan—the communal sharing of labour, which is practised widely among MASIPAG farmers.

While the network’s farmer-led approach leads to a high degree of internal connectivity, connectivity with external actors along the greater value chain is limited. Longstanding POs may engage in collective local marketing efforts, but with the majority of seed and produce determined for farm use and direct household consumption, market contacts remain limited. This explains Heckelman et al. (2018) finding that local market access for MASIPAG farmers does not significantly differ from that of conventional farmers. Although MASIPAG focuses on small-scale farmers that produce limited surpluses, their overall contribution to the country’s food security is not to be dismissed (c.f. Altieri, 2004). Given that approximately half of the Filipino population lives in rural areas and primarily works in agriculture, smallholders contribute significantly to community food security and play a central role in ensuring local, culturally appropriate food production. Furthermore, the high level of local connectivity and the pooling of resources and labour is what enables many of these farmers to produce surpluses in the first place. Since MASIPAG’s network structure allows for easy and cost-efficient up-scaling, the share of Filipino farmers benefiting from the approach could be widely extended if the network received adequate political and financial support.

With most farm inputs being produced locally, the network is largely autonomous from supplies from international agribusiness. External inputs that are connected to global supply chains such as chemical pesticides, synthetic fertilizers or genetically modified varieties have no relevance to the network. One farmer stated: “We have experienced that we cannot find what we would like to buy in the market. […] But because I have seeds, I can continue my farming […]. And that is not affected by the fluctuations in the market or by the lack of supply of farm inputs” [Interviewee #5]. Hence, MASIPAG’s focus on local rather than global connection contributes to agroecological resilience by reducing vulnerability to external factors such as market shocks, price fluctuations and legal restrictions.

### Builds human capital and encourages reflective and shared learning

Learning from experiences, mobilizing social resources, and sharing knowledge allows for anticipating the future based on past experiences and for building up the capacity to react to diverse disturbances (Cabell & Oelofse, 2012; Frankenberger et al., 2013; Stockholm Resilience Centre, 2015). Especially farmer-led, collaborative research has been identified as a central component of resilient seed and agricultural systems (c.f. McDonald et al., 2019; Waters-Bayer et al., 2015). Capacity building and collective learning are central components of MASIPAG’s work. POs that join the network undergo a series of hands-on training. An introduction to organic farming is followed by training on integrated diversified farming systems as well as by training on seed and variety collection, identification, maintenance, multiplication and evaluation. Advanced training modules include breeding, marketing, and advocacy. The training is delivered by over 100 volunteer farmer-trainers, who are practitioners themselves and familiar with the context-specific challenges faced by local farmers. With about 10 main languages and more than 150 local languages spoken in the Philippines, when possible, trainers are selected to provide culturally and linguistically sensitive teaching. One farmer described:

We gained respect and support when we [joined] MASIPAG. The knowledge that we got is priceless. Money can be lost, but knowledge is life skills. Through training, my knowledge and skills were enhanced and my farming system was improved and I came to adopt the ways of organic farming. And I know now how to take care of the seeds and use the local resources. […] Also, before I [did] not practice Bayanihan or helping other farmers, […] but now, we practice helping each other and I came to believe that my fellow farmers [are] my responsibility [Interviewee #5].

In addition to formal trainings, MASIPAG fosters a culture of shared learning through actively creating spaces and opportunities for knowledge exchange and experimentation at different organizational levels. These include farm visits, staff exchanges, and field days for collective variety selection. Moreover, PCBs follow a rotational schedule, whereby each meeting takes place at a different farm, to encourage on-site demonstrations and ensure farmers’ exposure to different farming practices. In addition, regular breeders’ forums at the regional and national levels facilitate knowledge exchange between farmers engaged in breeding and allow for feedback on breeding projects.

MASIPAG takes a holistic approach to learning and encourages creative expression and experimentation to develop active farmer-scientists, rather than passive recipients of technologies. To help farmers evaluate and improve their own inventions, MASIPAG runs a programme on farmer-developed and adapted technologies, through which farmers and scientists exchange knowledge and ideas during the development phase of new practices and technologies. Based on the belief that farmers are fully capable of developing and maintaining their own cultivars, the network aims to challenge the widespread assumption, that breeding can only be carried out by highly educated scientists. MASIPAG hence puts place-based environmental learning from observation and experimentation at the centre of knowledge generation. These findings are in line with research on farmer’s movements across the world that disproves the notion that farmers know little about their local agroecosystem and that technological fixes and inputs based on highly scientific “expert” knowledge are preferable to locally developed varieties and agricultural practices (Davila et al., 2018; Frossard, 2002; Šūmane et al., 2018). Technological innovations emerging from farmer-led research are primarily disseminated through informal spaces, have a high uptake rate by other farmers and can lead to substantial livelihood improvements (Waters-Bayer et al., 2015). Besides agricultural skills, the network aims to cultivate (political) leadership, critical thinking, and interpersonal skills, emphasizing the importance of farmers’ personal development for developing solution-oriented approaches and adaptive capacities. Social learning can thereby play a crucial role in adapting new practices and technologies (Chaudhuri et al., 2021). MASIPAG’s approach is also in line with the Inclusive and Sustainable Agricultural and Rural Development Model which among other things highlights the importance of farmer-to-farmer knowledge sharing for enhancing the capacity of communities to build effective agricultural systems that benefit poor and vulnerable groups (SEARCA, 2022).

Generating new knowledge on varieties and the socio-ecological systems they are embedded in requires continuous monitoring and documentation. To make accurate and long-term observations, record-keeping on agronomic characteristics is a key element in MASIPAG’s documentation practices and the network’s farmers have been found to keep better records than their conventional counterparts (Heckelman et al., 2018). MASIPAG farmers use “simplified evaluations sheets” to document their observations during trials and plantings and make them available to the network for further analysis. However, some interviewees reported challenges in accurately filling in these forms and suggested further simplifying documentation processes. Overall, MASIPAG’s farmer-to-farmer approach to knowledge transmission, its culture of shared learning and its active creation of spaces for experimentation may contribute to agroecological resilience by preparing farmers to respond creatively to diverse challenges.

### Conservative innovation that honours legacy

Systems that embody biological and cultural memory through honouring legacy address the challenges of future uncertainties by drawing on past conditions and experiences. This can increase their adaptive capacities (Cabell & Oelofse, 2012; Worstell & Green, 2017). MASIPAG honours legacy in several ways. The network considers seeds not as a product of a single breeder but as an outcome of the collective efforts of many farming communities that collected, propagated, and selected germplasm over thousands of years. MASIPAG acknowledges and celebrates the cultural heritage of seeds and considers their conservation for current and future generations to be a collective responsibility of farmers and breeders. Seeds are regarded as sacred, and their stewardship is hence a priority at all organizational levels.

One of MASIPAG’s main objectives is to preserve and improve traditional varieties. The majority of parental breeding material stems from traditional varieties and the continuous cultivation of these varieties is a matter of course for the network. Furthermore, traditional practices and knowledge, including indigenous knowledge, are integrated into breeding and seed production. For instance, traditional practices of threshing and drying seeds, as well as traditional breeding practices of crossing and selection are widely used by MASIPAG members. These low-tech practices can be easily used by resource-poor farmers and connect them to their heritage. A farmer stated:

Traditional practices in breeding [are] very, very helpful […] because [they] can be easily adapted and replicated by farmers on their own farms, unlike the breeding technologies that are done by the private institutions. With traditional practice […] the inputs you need are readily available and locally available. They are around you and connect you to the land and our heritage. [Interviewee #12]

Heckelman et al. (2018) also observed a higher level of traditional activities and knowledge preservation among MASIPAG farmers than among conventional farmers. The networks’ focus on traditional varieties and practices hence contributes to agroecological resilience by tapping into the genetic legacy and traditional wisdom that is indispensable in the development of adaptive varieties.

### Reasonably profitable

Reasonable profitability allows farmers and breeders to save for future investments and build up financial buffers in times of hardship and unexpected disturbances (Cabell & Oelofse, 2012). I distinguish here between the profitability of breeding and seed multiplication and the profitability of farming. Based on the belief that seeds are sacred, MASIPAG follows the principle that seeds are not to be sold, commercialized, or commodified in any way. Within the network, seeds are shared and exchanged free of charge. Seed multiplication is thus not a source of income generation. One staff member described: “We always believe that seeds [are] free. We don't sell the seeds, they are sacred. Even if you are the breeder of this particular variety or you are the producer of this particular seed, you can't sell your seeds. But you can always [...] share [them] with other farmers for free” [Interviewee #1].

There is also no financial compensation for farmers engaging in breeding efforts. Instead, social rewards such as recognition and respect by other MASIPAG members play an important role in incentivizing farmer-breeders. It is noteworthy, that MASIPAG’s approach allows even resource-poor farmers to engage in the time-consuming and labour-intensive activities of breeding. Variety development in the Philippines is predominantly carried out by large-scale research institutes that are well-resourced, often funded through government support, and have high investment volumes. Developing and testing a high-yield variety and making it available to farmers is a costly and lengthy process that often takes more than a decade. Contrasting, MASIPAG's farmer-breeders develop new varieties at essentially no additional costs and in shorter timeframes, since breeding activities are integrated into the day-to-day operations of farmers and new varieties do not have to undergo a lengthy registration process.

While breeding and seed production do not provide income for farmers, they may significantly reduce farmers’ input costs. One of MASIPAG’s primary aims is to enable farmers to achieve self-sufficiency in their production, which has been associated with positive impacts on yield, economic benefits and quality of life (Abas, 2016). Farm input costs for rice production in the Philippines are some of the highest in the region. Average input costs of conventional rice production across seasons were at 12.41 PHP/kg [22 US cents/kg]—including 2.88 PHP/kg [5 US cents/kg] for seeds and agrochemicals and 4.42 PHP/kg [8 US cents/kg] for labour costs before the COVID-19 pandemic hit (Bordey et al., 2016). With an annual inflation rate of over two per cent in 2020 and 2021, they have likely increased significantly, as have overall food prices. The cultivation of hybrid varieties requires high levels of overall investment that may not be economical for cultivation in the wet season, dry-season cultivation without irrigation, or low-lying plots of land (Glover et al., 2020). By relying on farmer-saved seeds rather than costly hybrid or certified seeds, replacing synthetic fertilizers and chemical pesticides with low- to no-cost organic fertilizers and natural pest management, and employing the labour-sharing principle of Bayanihan, MASIPAG farmers can substantially lower input costs. A farmer described:

We have evaluated the 10-year program implementation of MASIPAG. […] We found out that in terms of income [MASIPAG] farmers are way better off than their conventional counterparts […] because we are cost-effective and have reduced the input costs. […] Here in the Philippines, once the [conventional] farmer planted their crops, they already had a lot of investments and they are in dept. The idea of MASIPAG is to produce your own inputs on the field, so you can farm with minimum expense [Interviewee #8].

MASIPAG’s primary focus is not on increasing income or profit, but rather on ensuring the food security of its members and their communities. However, 74 POs engage in joint local marketing of their produce, which is certified by the network’s participatory guarantee system for organic production that has been in place since 2005. This system provides an affordable alternative to costly third-party certification that many farmers cannot afford and is recognized by the Filipino government since December 2019. Despite the adaption of the Filipino National Organic Agricultural Act in 2010, certified organic farming remains a niche market constituting only 1.6% of the country’s agricultural production (Willer & Lernoud, 2019). However, it is likely that a large share of organic produce is not officially certified and does not count towards these statistics. Local organic produce has a high cross-price elasticity (Digal & Placencia, 2020), but a price premium for local organic rice and heirloom varieties applies (Glover et al., 2020; Velasco, 2019). Direct marketing hence significantly increases the revenues of MASIPAG farmers that can sell their surplus. As a rising number of MASIPAG’s POs professionalize and are able to up-scale their production, certification through the participatory guarantee system becomes increasingly attractive for farmers.

There is mixed evidence regarding the overall profitability of MASIPAG farmers. Based on a survey of 840 farmers, Bachmann et al. (2009) find the net agricultural income per hectare on average to be 66% higher for MASIPAG farmers compared to conventional farmers and report even larger benefits when produce for subsistence is included in calculations. In contrast, based on a sample of 40 smallholder farmers in Negros Occidental, Heckelman et al. (2018) find no statistically significant difference between MASIPAG and conventional farmers in several profitability indicators. They also observe an overall low level of financial savings and a level of high reliance on additional financial support among both conventional and MASIPAG farmers. In summary, MASIPAG’s principles on the non-commercialization of seeds and varieties, do not allow for income generation, but the initiative’s approach may help farmers reduce input costs and enable them to engage in seed work.

### Bases on polycentric, decentralized governance structures

Polycentric governance systems are systems with multiple governing authorities at varying scales (Ostrom, 2010). They foster resilience by creating modular and functional redundancy of governing structures that can maintain crucial system elements in times of disturbance (Biggs et al., 2015; Stockholm Resilience Centre, 2015). Through its reliance on organizationally and financially independent farmer groups (POs) in 63 out of 82 Filipino provinces, MASIPAG’s governance structures are fully decentralized (see Fig. 1). POs are encouraged to adopt democratic decision-making structures and elected farmer-leaders represent POs at all organizational levels. To ensure that farmers’ needs are at the centre of all decision-making, farmer representatives outnumber non-farmer members (staff, NGO representatives and scientists) in all of the network’s committees. MASIPAG’s participatory guarantee system also follows a decentralized approach, with farmer committees inspecting and attesting other farmers’ adherence to organic breeding and cultivation practices.

This decentralized, bottom-up approach allows for quick responses to the needs of the lowest organizational levels, including during times of disturbance. It also fosters farmers’ sense of responsibility for their community, helps them to coordinate responses to the multiple stresses of the environment and strengthens place-based knowledge and resources generation. A board member stated:

The key is decentralization, because […] we are a widely dispersed network, working in over 60 provinces, with limited staff. It’s not so easy to coordinate […] so there must be some degree of decentralization […]. We are organised in local groups and to me, that’s the most critical aspect because we can quickly respond to the needs and priorities of the lowest level. It’s very efficient [Interviewee #2].

This form of smallholder empowerment, embedded in localized social networks that act as independent decision-making entities, fosters the democratization of agriculture and ultimately food sovereignty (Heckelman, 2019). It can however also lead to conflicts on power and resource distribution and relies on farmers’ willingness to have a high level of engagement with the network. Interviewees for example reported that farmers of the regional cluster in Mindanao have striven for organizational independence from the nationwide network due to internal conflicts. The level of farmers’ involvement with the network also varies significantly, with some farmers only being peripherally involved, which weakens the social cohesion that is central to the functionality of the network. Overall, MASIPAG’s polycentric organizational approach contributes to agroecological resilience through the proliferation of responsibility, the ability to attend flexibly to localized needs, and the redundancy in governance structures. However, internal governance disputes may undermine resilience since they have the potential to fragment governance structures and lead to power inequalities.

### Ensures resource access and broadens participation

Resource access and broad participation contribute to agroecological resilience by facilitating the collective action required to respond to disturbances (Biggs et al., 2015; Stockholm Resilience Centre, 2015). Access to seeds and genetic resources is a priority for MASIPAG. The network believes that farmers’ control over seeds, genetic resources and technologies helps them to regain confidence and reassert control over their production system. They hence promote the rights of farmers to (i) use, save, exchange, and multiply seeds without restriction, (ii) access seeds and genetic resources that are appropriate to local environments as well as to farmers’ capacities and needs, and (iii) freely choose the seeds they use (MASIPAG Statement on farmers’ rights published by GRAIN, 2002).

All seeds are freely shared within the network and with other community members once they have received an introduction to the network’s principles and values regarding seed handling. Farmers and breeders are considered to be stewards not owners of genetic resources and any form of intellectual property rights such as patents or variety protection is rejected. Privatization of genetic resources that could in any way limit farmers’ access to genetic material is seen as contradicting the very essence of farmers’ rights, which build on collective rights and responsibilities. Although MASIPAG’s approach to seed governance is based on common ownership, the network does not support unrestricted access. MASIPAG sees a need to protect its varieties from appropriation and commodification through the private seed sector and does not share its varieties with private seed companies or government-supported large-scale research institutes such as the International Rice Research Institute (IRRI) or the Philippine Rice Research Institute (PhilRice). For the same reasons, MASIPAG chooses not to register its varieties with the Filipino Community Seed Registry and does not collaborate with the National Seed Industry Council. These restrictions may limit the widespread use of MASIPAG’s varieties and isolate MASIPAG in the long term, but the network believes that they are necessary to ensure the long-term survival of MASIPAG as an independent, bottom-up initiative.

Participation in breeding and seed production is broadened through MASIPAG’s focus on farmers’ self-sufficiency. Although MASIPAG’s early breeding activities were predominately carried out by its scientist members, participatory breeding approaches were gradually employed to include farmers’ perspectives and preferences and build up their breeding knowledge. Following its focus on farmer empowerment, the network has now fully shifted to farmer-led breeding. Farmer-breeders carry out all steps of the breeding process: They set their own breeding objectives, select parental material, cross-pollinate, select lines, and evaluate their selections. This is considered to be an act of political resistance. One board member described:

The socio-political dimension of breeding is a manifestation of what we are calling farmers’ empowerment. That [the farmers] can feel the pride in themselves, in being able to create a new variety. […] It’s a tool of farmers’ empowerment, because, at a time when private corporations are patenting seeds […] if the capacity to develop and maintain seeds is brought back to the farmers, they can survive without money to buy seeds. They can have their own seeds. From the technical side, of course, breeding creates more biodiversity [and farmers] can develop varieties that will suit their specific objectives: better eating quality, more adapted to environmental stress, like flooding, salt-water tolerance. […] These are all immediate objectives, but the political objective behind that is to make sure that the capacity to maintain and improve the seeds is right there in the hands of those who are most in need: the farmers [Interviewee #2].

In line with findings from Silva Garzón & Gutiérrez Escobar (2019), agroecological farmers engaging in seed work, often view their actions as a form of resistance that is mobilized through narratives of sovereignty, solidarity, environmental protection, tradition and freedom. All of these narratives were addressed in the interviews and point to the politicization of seed saving to achieve larger aims of food sovereignty and the preservation of identity and autonomy. However, with 82 farmer-breeders to date—70 rice breeders and 12 corn breeders—only a small percentage of the networks’ farmers have become active in variety development. The corn breeding programme was also only recently revived after it was suspended in 1998 due to being too technical. Nevertheless, according to Bachmann et al. (2010) over three-quarters of MASIPAG farmers practice seed selection, compared to a quarter of conventional farmers. Hence, MASIPAG’s focus on self-sufficiency and farmer control over seeds and breeding contributes to agroecological resilience by broadening the base of actors engaged in the stewardship of genetic resources. However, it is noteworthy that despite the extensive breeding programme, the majority of MASIPAG varieties were developed by only relatively few breeders.

### Encourages complex adaptive systems thinking

Complex adaptive systems thinking entails fostering holistic and integrative rather than reductionist approaches, including recognizing the importance of slow variables, uncertainties, and complex system dynamics (Biggs et al., 2015; Stockholm Resilience Centre, 2015). MASIPAG strives to understand the complexities of local economic, environmental, and sociocultural realities, including farmers’ role and agency in stewarding genetic resources. The network acknowledges that farmers are dependent on seeds, as much as seeds are dependent on farmers and translates this egalitarian worldview of working with nature (c.f. Douglas, 1978; Thompson et al., 1990) into a holistic approach to breeding and seed production. Embracing seeds’ cultural embeddedness and ecological relevance, MASIPAG adapts a “no regrets” approach to risk management and has developed a climate change resilience programme to actively build up its resilience capacities.

Furthermore, in recognition of the complex dynamics of social-ecological systems, social-organizational and technical solutions are given equal weight in programme design and problem-solving. MASIPAG as an institution sees its work in the context of greater global power dynamics and a growing influence of agrochemical companies that focus on technology-oriented solutions rather than social innovations. A board member reported:

Climate change is worsening and we are narrowing the genetic pool. […] We are putting chemical fertilizers and pesticides for creating an ideal environment for the crop to grow. That kind of farming is more vulnerable to disruptions. […] We need to factor in sustainability and equality, not just profit. These are the big things to consider because the dominant paradigm and those who are in power are more under the influence of profit - at the expense of the environment. We might have a higher yield right now, but what is the cost of pollution? There are trade-offs, so we have to look at other system states. The parameter of looking at things in the world [should] not only be efficiency, but also sustainability. […] Other organisations often only focus on the technical solutions, MASIPAG also looks at social solutions: sharing of seeds and technology, sharing of work, cooperation, collective planning and decision-making [Interviewee #2].

The network strives for broad political change through collective action and has come to be a focal point for political mobilization, advocacy, and campaigning, for instance through engaging in rallies about genetically modified crops, land reforms and other matters. Considering current trends of further agricultural intensification, MASIPAG does not oppose progress, or dismiss the relevance of technology or science, but has with time developed its own definition of progress that is centred around Filipino farmers’ rights and needs and based on a profound understanding of local social-ecological system interactions. This understanding of progress highlights the need for strengthening resilience as a primary objective. The findings emphasize the importance of locally rooted social networks and collective action that strengthen seed and food sovereignty by empowering farmers to define their own culturally and environmentally appropriate practices (c.f. Chaudhuri et al., 2021; López et al., 2019; Porcuna-Ferrer et al., 2020; Silva Garzón & Gutiérrez Escobar, 2019).

Despite the networks’ holistic approach and collective action, it has so far not destabilized the current predominant agricultural regime. Yet, regime destabilization is a central aspect of promoting and speeding-up sustainability transitions (Kanger et al., 2020). While the network has been effective in rallying against genetically modified organisms and achieving official recognition of its participatory certification system, the comprehensive political change envisioned by MASIPAG requires broader political alliances, which the network has so far not been able to establish. Such political support would have to focus on both the scaling-up of MASIPAG’s approach as well as on challenging predominant business-centric agricultural development paradigms focused on output maximation and technocratic approaches. To conclude, MASIPAG fosters agroecological resilience by adapting a holistic breeding approach and taking into consideration the complex interrelations and dynamics of local and global social-ecological systems in programme development and strategic matters. However, its ability to contribute to larger regime destabilization is hampered by a lack of broader political support.

Table 2 summarizes the contribution of MASIPAG’s commons-based approach to breeding and seed production on agroecological resilience and outlines aspects that pose disadvantages or may threaten the longevity of the network.

**Table 2:** Contributi﻿on of MASIPAG’s commons-based approach to breeding and seed production on agroecolog﻿ical resilience as well as disadvantages of the networks’ approach

Indicator for agroecological resilience	Characteristics of MASIPAG’s commons-based approach to breeding and seed production	Aspects that pose disadvantages or may threaten the longevity of the network
Socially self-organized	Bottom-up network with a high degree of self-organization among farmers, which is a prerequisite for gaining membership status. Farmer-led committees at all organizational levels ensure collective seed stewardship.	Non-cooperation between farmers, due to conflict or disputes may hamper resilience capacities. Social self-organization presupposes a high level of engagement of farmers.
Ecologically self-regulated	Varieties are bred specifically for organic cultivation conditions. Only open-pollinated, naturally reproducible varieties are bred and cultivated. Hybrid varieties and genetically modified varieties with high-input requirements are rejected	Open-pollinated varieties typically have lower yields under optimally controlled environmental conditions.
Functional and response diversity	The breeding of open-pollinated varieties favours high genetic diversity. The in situ maintenance of conservation varieties enhances agrobiodiversity. Trial and backup farms continuously ensure high levels of diversity of rice varieties. Crop diversification at the farm level is encouraged.	
Optimally redundant	A network of trial and backup farms ensures the continuous cultivation of all varieties at multiple locations throughout the country. Farmers are encouraged to build up seed reserves.	Seed reserves are not build up by all MASIPAG farmers. Backup mechanisms may not function well if entire regions are affected by calamities.
Exposed to disturbance	Variety development and selection are carried out on-farm to ensure exposure and adaptation to local geophysical and environmental conditions.	
Coupled with local natural capital	Farm inputs are generated on-farm or sourced locally. Varieties are bred for and trialled based on their adaptation to local environmental conditions.	Heavy reliance on local resources increases vulnerabilities in case of local resource depletion that is beyond the networks’ control.
Appropriately connected: globally autonomous and locally interdependent in modular subsystems	A high degree of local connectedness for instance through labour-sharing practices and regular seed exchanges among farmers is ensured. Famers strive for almost complete independence from external inputs.	
Builds human capital and encourages reflective and shared learning	Capacity building is ensured through training in organic seed production, breeding and marketing, delivered by farmer-trainers. Shared learning is fostered through farm exchanges, breeders’ forums and thematic committees that enable knowledge exchange at all organizational levels. Experimentation by active farmer-scientists is encouraged.	
Conservative innovation that honours legacy	Seeds are considered sacred, and the cultural heritage of seeds is actively acknowledged and celebrated. Traditional and indigenous practices and knowledge is integrated into seed production, including the use and further development of traditional breeding techniques.	
Reasonably profitable	Self-sufficiency in the production of seeds and other farm inputs may substantially reduce farmers’ input costs.	Breeding and seed production do not allow for income generation.
Bases on polycentric, decentralized governance structures	Organizationally and financially independent farmer groups lead to decentralized decision-making structures that allow for quick responses to local needs. A decentralized participatory guarantee system ensures quality control.	Internal governance disputes may fragment governance structures and undermine resilience.
Ensures resource access and broadens participation	Farmers’ control over seeds is a central priority, but breeders and farmers are considered stewards, not owners of varieties. Any form of privatization of seeds, including intellectual property rights, is rejected. Seeds are shared freely and at no cost within the network and with community members who agree to MASIPAG’s principles. Varieties are developed by farmer-breeders and all farmers are encouraged to practise seed saving.	Relatively few farmers engage in breeding. Refusal to register varieties with the Community Seed Registry and lack of cooperation with the National Seed Council may isolate the network in the long term.
Encourages complex adaptive systems thinking	An egalitarian worldview of working with nature leads to a holistic approach to breeding and seed production that takes into account the complex local economic, environmental, and social realities. Social-organizational and technical solutions are given equal weight in problem-solving and programme design.	Limited regime destabilization, due to a lack of broader political support.

## Conclusion

On the example of the Filipino farmers' network MASIPAG, this paper examines the relationship between commons-based breeding and seed production and agroecological resilience. MASIPAG’s primary aim to alleviate poverty and increase food security is achieved by empowering resource-poor farmers, through access and control over production means, including genetic resources. Seed work is thereby at the centre of the network’s activities, and deeply ingrained in the network’s culture. As one farmer described it*:* “The seeds act like the social, cultural and material glue that forms the foundation of the network” [Interviewee #8]. They are regarded as a sacred common good of cultural significance, rather than as a commodity.

The commons orientation of the network's approach to breeding and seed production through joint resource governance, collective responsibility for the provision and development of varieties and polycentric organizational structures contribute to building agroecological resilience capacities in various ways: By equipping small-scale farmers with the tools to regain control over seed production and breeding, they become stewards of an actively evolving collection of varieties. The in situ maintenance of traditional varieties, an elaborate network of diversified trial and backup farms and the continuous maintenance of seed reserves lead to the build-up of buffering capacities. A holistic approach to the breeding of open-pollinated varieties that are suited to organic cultivation conditions, exposed to broad environmental interactions, and trialled throughout the country, fosters plant robustness, local adaptation, and agrobiodiversity conservation. A focus on regionally available natural resources and almost complete independence from external inputs and global agricultural value chains reduce vulnerabilities to external factors. Adaptive capacities are strengthened through a high degree of flexibility and responsiveness achieved by farmer-led self-organization from the local to national level. Farmers’ capacities to respond to diverse challenges are further built by creating spaces for experimentation, farmer-led training and research, and through fostering a culture of shared learning.

However, the networks’ approach also bears several risks that may negatively affect smallholders’ resilience capacities. The strong reliance on self-organization presupposes a high level of engagement of farmers which not all farmers are able or willing to provide and thereby excludes smallholders that are not able to engage in communal efforts. Furthermore, internal conflicts and governance disputes may lead to non-cooperation and power imbalances that pose a risk to the network’s functioning as social insurance. Moreover, the heavy reliance on local resources makes MASIPAG farmers vulnerable to local resource depletion that is beyond the networks’ control. MASIPAG’s hesitation to share its seeds with commercial and governmental actors may limit the widespread adaption of its varieties and may isolate the network in the long term. Protection of its varieties may, however, be necessary to limit unjustified appropriation by actors with commercial interests. The network also scores relatively low on profitability since MASIPAG’s principles on the non-commercialization of seeds limit possibilities for income generation. Even so, the network's approach allows for cost-effective breeding and reduces farmers’ input costs, which may positively affect their financial situation.

Overall, the case study demonstrates the relevance of commons-based approaches in building agroecological resilience. It highlights the potential of empowering farmers to define their own culturally and environmentally appropriate practices to improve seed and food sovereignty and points to the role of farmer-led, collaborative research for building seed and agricultural systems that foster agrobiodiversity. Furthermore, the analysis emphasizes the adaptive capacities of organic farming and breeding.

By turning seeds into anti-commodities, commons-based seed initiatives can protect local interests and cultural values. However, since such initiatives often provide services with relatively low visibility, conventional economic measures and policymakers may easily overlook the value of their seed work in strengthening agroecological resilience and ultimately food security. Most policy incentives in the Philippines continue to be geared towards cash crops such as sugarcane, corn, coconuts, pineapples, and bananas, produced by farmers that are passive recipients of knowledge and technology, with little attention paid to diversified livelihoods, agrobiodiversity, or climate change vulnerabilities. Commons-based networks such as MASIPAG hence provide alternatives to the predominant agricultural development paradigms but require the necessary resources and political alliances to challenge the biohegemonic structures of industrial agriculture. A reorientation of Filipino seed and agricultural policies geared towards strengthening agroecological resilience would be a first step in reducing farmers’ vulnerability to social, economic, and environmental disturbances. Such a policy shift would increase the adaptative capacities of the Filipino agricultural sector to future climate change impacts and thereby secure long-term food security and livelihoods.

## Data Availability

The datasets generated and analysed during the current study are available from the corresponding author on reasonable request.
